# Effect of Flat Running Shoes on Hip Kinematics in Male Recreational Runners

**DOI:** 10.3390/ijerph192416473

**Published:** 2022-12-08

**Authors:** Masen Zhang, Jing Cui, Hui Liu

**Affiliations:** 1 Biomechanics Laboratory, School of Sport Science, Beijing Sport University, Beijing 100084, China; 2 China Institute of Sport and Health Science, Beijing Sport University, Beijing 100084, China

**Keywords:** biomechanics, shoes, iliotibial band syndrome, patellofemoral joint pain, statistical parametric mapping

## Abstract

Patellofemoral joint pain and iliotibial band syndrome are very common running−related injuries. Excessive contralateral pelvic drop, hip adduction, and hip internal rotation have been suggested to be associated with the two injuries. The purpose of this repeated measures and the cross−sectional study was to investigate the effect of flat running shoes on these kinematic variables compared with that of conventional running shoes with a 10 mm drop. Eighteen male recreational runners were recruited to run in flat shoes and conventional shoes with a 10 mm drop, in random order. Impact force data and lower extremity kinematics were synchronously obtained using two Kistler force plates and eight motion infrared cameras, whereas differences in the impact force and hip kinematics were compared using statistical parametric mapping. Regarding hip kinematics, the hip flexion (*p* = 0.004) and adduction angles (*p* = 0.004) decreased significantly at 30–70% and 62–85% of the stance phase, respectively, while wearing flat running shoes; the contralateral pelvic drop angle (*p* = 0.001) decreased significantly at 31–75% of the stance phase while wearing flat running shoes. The knee internal rotation angle (*p* = 0.035) decreased significantly at 8–17% of the stance phase while wearing flat running shoes compared with conventional running shoes. Given that these kinematic variables are associated with patellofemoral joint pain and iliotibial band syndrome, flat running shoes may have potential benefits for the prevention or treatment of knee injuries.

## 1. Introduction

Running is an effective and popular physical activity known to be easily implemented with minimal equipment and improve cardiovascular function, physical fitness, and mental health [1]. However, up to 79% of runners suffer from running−related injuries annually, leading to social and economic losses [2]. The knee is the most frequently injured joint affecting runners at all distances, with patellofemoral joint pain (PFP) and iliotibial band syndrome (ITBS) being very common knee injuries. There are indications that these injuries influence the quality of life by resulting in serious medical conditions and sometimes even osteoarthritis [3,4].

Several proximal biomechanical factors have been proposed as potential contributors to both PFP and ITBS, with a special focus on hip strength and hip kinematics. A study has shown that stronger pre−injury hip abduction and weaker pre−injury external rotation strength are associated with the development of PFP [5]. Other studies suggest that reduced hip strength is a result of PFP rather than the cause [6,7]. Prospective studies have found that runners who later developed PFP exhibited a greater hip adduction angle and internal rotation angle [8,9]. Additionally, a systematic review found moderate evidence that runners with PFP also had increased hip adduction and contralateral pelvic drop [10]. Elevated patellofemoral joint stress has been demonstrated as a mechanism of PFP, and the increases in contralateral pelvic drop, hip adduction, and hip internal rotation result in the lateral displacement of the patella relative to the femur, reducing the contact area of the patellofemoral joint, and increasing the force and stress of the lateral patellofemoral joint on the subchondral bone, thereby resulting in pain [11,12,13,14]. Gait retraining in subjects with PFP resulted in a significant reduction in pain and improvements in function following a reduction in the hip adduction angle and contralateral pelvic drop [15,16].

Excessive contralateral pelvic drop, hip adduction, hip internal rotation, and knee internal rotation are also associated with ITBS [17,18,19,20]. Excessive contralateral pelvic drop refers to the improper leveling of the pelvis, which causes one side of the hip to drop lower than the other during running or any other activity that requires single−leg support. Contralateral pelvic drop increases due to reduced hip abduction strength on the standing side of runners with ITBS [21,22]. Prospective studies found that runners at risk of ITBS showed greater hip adduction and knee internal rotation [17,18]; additionally, runners with ITBS demonstrated greater hip adduction, hip internal rotation, and knee internal rotation compared to healthy controls [19,20]. The dynamic valgus movement of the distal femur and the knee joint was reported to increase the strain on the iliotibial band and compress the abundantly innervated fat pad above and below the lateral femoral condyle, thereby resulting in pain [17,22].

Excessive contralateral pelvic drop, hip adduction, hip internal rotation, and knee internal rotation are associated with PFP and ITBS; reducing the value of these factors is therefore considered a strategy for the prevention and treatment of PFP and ITBS [8,9,17,18,23,24]. While strengthening the hip abductor muscles may alleviate the symptoms, only hip strength training is not sufficient to alter kinematics [25]. Recently, gait retraining has demonstrated positive effects on clinical and functional outcomes [15,26,27,28]. Changes in the biomechanics of the hip and knee joints were also reported with the alternation of strike patterns and running shoes [29,30,31,32].

An important feature of conventional running shoes is the heel−to−toe drop (i.e., the thickness difference between the forefoot and heel parts of the sole) of approximately 10 mm, which may be related to the high rate of knee injuries [29,33]. Sports product companies have introduced running shoes with heel−to−toe drops to reduce the peak magnitude of impact force and provide better cushioning. Running shoes with heel drops have no effect on the vertical impact force, according to studies, while also causing a rearfoot strike pattern and greater loading on the knee joint [29,32,33,34,35,36]. Running shoes with drops equal to or greater than 6 mm resulted in a greater foot inclination angle on initial contact [32,36] and increased the knee extension moment and patellofemoral joint loading compared with running in minimalist shoes (with drops equal to or close to 0 mm) or barefoot [29,33,34,35].

Despite acknowledging that hip kinematics contribute to knee injuries, a lack of research exists regarding the influence of running shoes with and without drops on three−dimensional hip kinematics. One study demonstrated that running in conventional shoes increased the peak joint reaction force at the hip joint [37]; other studies showed no significant differences in the biomechanics of the hip joint while wearing conventional shoes with drops compared to minimalist shoes and being barefoot [38,39]. The different results of previous studies are likely due to using different kinds of running shoes with varying materials and constructions; additionally, different types of minimalist shoes induced different changes [40]. To determine the effect of flat running shoes without a drop on proximal kinematics, it is necessary to compare shoes that only vary in heel−to−toe drop and thereby clarify the differences in hip kinematics related to PFP and ITBS.

The purpose of this study was to investigate the effect of flat running shoes on hip kinematics in comparison with that of conventional running shoes with a 10 mm drop and further discuss the potential effects on PFP and ITBS. We hypothesized that contralateral pelvic drop, hip adduction, hip internal rotation, and knee internal rotation would decrease while wearing flat running shoes.

## 2. Materials and Methods

### 2.1. Participants

Based on the effects of risk factors in a prospective study [17], a sample of thirteen participants was required to provide a statistical advantage for the study (alpha of 0.05 and power of 0.8). Eighteen male recreational runners were recruited to participate in this repeated measures and cross−sectional study [41]. The participants had a mean (SD) age of 23.1 (1.6) years, a height of 1.74 (0.04) m, and a body mass of 65.0 (3.9) kg. The three inclusion criteria for participation were: running with a rearfoot striking pattern, confirmed by video graphic analysis [42]; running for at least three times a week [41]; and having no musculoskeletal or neurological injuries in the six months before the trials. All participants signed the documented consent accepted by the Ethical Institutional Review Board of Beijing Sport University (2020133H).

### 2.2. Procedures

Participants performed a 10 min warm−up before evaluation to adapt to the test shoes. Reflective markers were placed on the right and left anterior iliac spines, anterior aspect of the thigh, lateral and medial condyles of the femur, anterior superior tibia, lateral and medial malleolus, calcaneus, and middle of the second and third metatarsal heads. Additionally, a marker was placed at the posterior iliac. After the participants underwent static calibration, the medial markers were removed.

**Figure 1:**
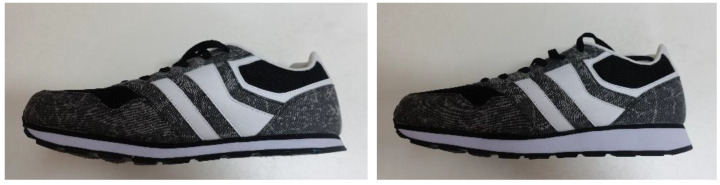
Flat running shoes and conventional running shoes with a 10 mm drop.

Running trials were conducted on a 30 m indoor running track at the Biomechanics Laboratory of Beijing Sport University. First, the marker−based motion analysis system was calibrated, and the test can only be started when the error of 500 mm between the two markers is less than 0.2 mm. Participants were required to run wearing flat running shoes and conventional running shoes with a 10 mm drop, in random order. Both shoes have the same structure and appearance, and the soles are made of ethylene vinyl acetate (EVA) and rubber with the same thickness in the forefoot part of 12 mm (Figure 1). Participants performed three overground running trials for each shoe condition; trials were accepted if the speed was 4.0 m/s (±5%), and right lower extremity data were collected [41].

### 2.3. Data Collection

**Figure 2:**
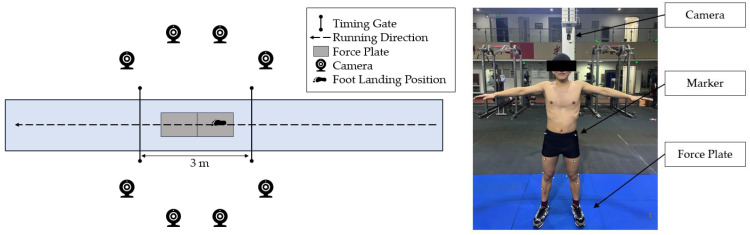
Experiment setup and marker placement.

Impact force data was obtained through 900 × 600 mm force plates (Kistler Instrument, Winterhur, Switzerland), sampling at 1000 Hz. Three−dimensional marker trajectories for the standing calibration and running trials were captured through an eight−video camera motion analysis system (Motion Analysis Inc., Santa Rosa, CA, USA), sampling at 200 Hz, which was considered the non−invasive gold standard of motion analysis and is widely used in biomechanical research [43,44,45]. The running speed was recorded using timing gates (Brower Timing Systems, Draper, UT, USA) located at both sides of the force plates with a spacing of 3 m (Figure 2).

### 2.4. Data Reduction

The marker trajectory data were filtered using a low−pass Butterworth filter with a cutoff frequency of 12 Hz [41]. The outcomes of this study were gait parameters, the three−dimensional angles of the hip and knee joints, and impact forces. Joint centers and three−dimensional joint angles were defined as recommended by the International Society of Biomechanics [32,46]. The contralateral pelvic drop angle was defined as the pelvis segment relative to the ground [47], whereas the foot inclination angle was defined as the angle between the long axis (the line connecting the calcaneus and the middle of the second and third metatarsal heads) of the foot and the ground upon initial contact. The vertical impact peak and vertical active peak values were the first and second maximum values of the vertical impact force, respectively.

### 2.5. Statistical Analysis

Gait and hip characteristic variables were compared between flat running shoes and conventional running shoes with a 10 mm drop using paired t−tests. The time series data for hip kinematics were compared using statistical parameter mapping (SPM) [48]. Statistical analyses were performed using SPSS 20.0 (SPSS, Chicago, IL, USA) and MATLAB R20116a (MathWorks, Natick, MA, USA). A type I error rate ≤ 0.05 was considered statistically significant.

## 3. Results

**Figure 3:**
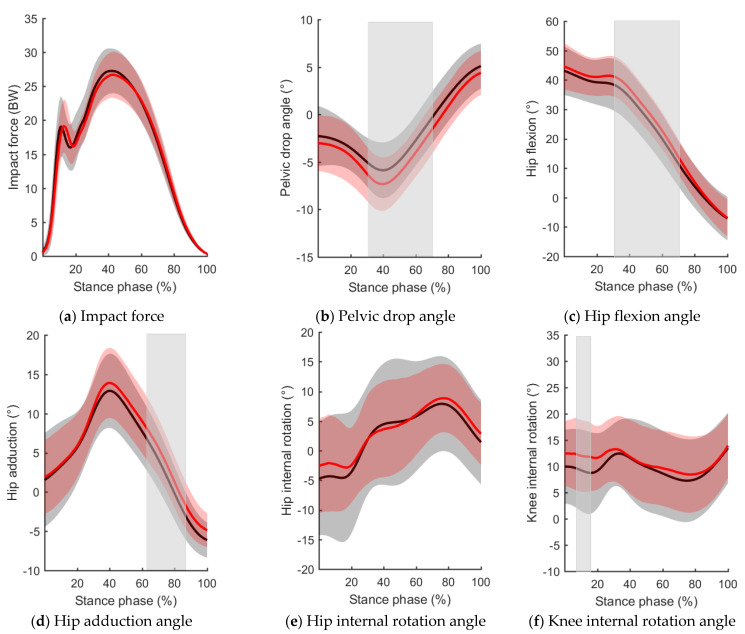
Comparison of impact force, hip kinematics, and knee internal rotation time series between flat shoes (black line) and conventional shoes with a 10 mm drop (red line): (**a**) impact force; (**b**) pelvic drop angle; (**c**) hip flexion angle; (**d**) hip adduction angle; (**e**) hip internal rotation angle; (**f**) knee internal rotation angle.

There were no significant differences in the step length, step frequency, vertical impact peak, vertical active peak, and impact force time series between flat shoes and conventional shoes with a 10 mm drop (Table 1, Figure 3). The foot inclination angle on initial contact decreased by 5.7° when wearing flat shoes compared with wearing conventional shoes with a 10 mm drop (*p* = 0.001, Cohen’s d = 0.71; Table 1). Regarding hip kinematics on initial contact and at the peak value, the peak contralateral pelvic drop was significantly lower when wearing flat shoes (*p* = 0.003, Cohen’s d = 0.51; Table 2). There were no significant differences between flat shoes and conventional shoes with a 10 mm drop for other variables (Table 2).

Regarding hip kinematics during the stance phase, compared with wearing conventional shoes with a 10 mm drop, the hip flexion (*p* = 0.004) and adduction angles (*p* = 0.004) decreased significantly at 30–70% and 62–85% of the stance phase, respectively, while wearing flat shoes. The contralateral pelvic drop angle (*p* = 0.001) decreased significantly at 31–75% of the stance phase while wearing flat shoes. Regarding knee kinematics during the stance phase, the knee internal rotation angle (*p* = 0.035) decreased significantly at 8–17% of the stance phase while wearing flat shoes compared with wearing conventional shoes with a 10 mm drop (Figure 3).

## 4. Discussion

The SPM results of this study partially support our hypothesis. Compared with conventional running shoes with a 10 mm drop, flat running shoes resulted in significant decreases in contralateral pelvic drop at 31–75% of the stance phase, significant decreases in hip adduction at 62–85% of the stance phase, and significant decreases in knee internal rotation at 8–17% of the stance phase, respectively. These may reduce the lateral displacement of the patella relative to the femur, increase the contact area of the patellofemoral joint, and reduce the force and stress of the lateral patellofemoral joint on the subchondral bone, thus relieving PFP [11,12,13,14]. Additionally, these may reduce the tension of the iliotibial band and the compression of the richly innervated fat pads above and below the lateral femoral condyle, thus relieving ITBS [17,22]. The findings of this study may provide valuable information for reducing these running−related knee injuries.

The results of the *t*−test demonstrated that with the exception of the decrease in the peak contralateral pelvic drop, no differences were detected between shoe conditions with respect to hip flexion, adduction, and internal rotation on initial contact and at the peak values. In line with our study, previous research also found no significant differences in hip kinematics on initial contact and at peak values between running in conventional shoes and minimalist shoes [38,39]. Other studies have reported that, compared with conventional shoes, barefoot running decreased the contralateral pelvic drop, hip adduction, hip internal rotation, and hip joint reaction force [37,49]. These results indicated that altering shoe structure had less of an effect on the hip joint than removing running shoes and was not sufficient to affect the characteristic values of hip kinematics. However, lower limb joint kinematics and kinetics change with time during running, and the traditional statistical analysis of zero−dimensional data ignores the sample order, resulting in focus bias and covariance bias [50].

Most running−related injuries are chronic, i.e., they relate to repeatedly overloading the musculoskeletal structures during running [51]. Regarding the risk factors for chronic injuries, the abnormal gait time series was more interpretive and meaningful than the characteristic values. SPM overcomes the above defects based on spatiotemporal smoothing and standardized data and applies the random field theory for a topological analysis in order to statistically analyze one−dimensional, continuous data [48,52]. SPM results showed that, compared with wearing conventional shoes with a 10 mm drop, the contralateral pelvic drop angle, hip flexion angle, hip adduction angle, and knee internal rotation angle decreased significantly during part of the stance phase while wearing flat shoes. These results are consistent with those of previous studies, which reported that although no differences were detected in peak data, barefoot running resulted in smaller contralateral pelvic drop, hip flexion, adduction, and internal rotation at 10% and 40% of the stance phase [49]. 

Differences in the foot inclination angle between the types of shoes may explain some of the differences in hip kinematics recorded in this study. The results demonstrated that when participants ran in flat shoes, the foot inclination angle decreased by 5.7° compared with those who ran in conventional shoes with a 10 mm drop. As the foot inclination angle decreased on initial contact, runners attempted to adopt a soft landing with more surface area, thus reducing acute pressure on the heel [40]. Previous studies reported that the rearfoot strike pattern resulted in significantly greater contralateral pelvic drop, hip adduction, knee abduction moment, and patellofemoral joint stress [53,54]. While wearing conventional shoes with a 10 mm drop, it is common for runners to strike the ground with more dorsiflexion and rearfoot area; this increases the horizontal distance between the supporting leg and the gravity center of the human body, which causes increases in the quadriceps muscle arm and knee extension moment. In other words, the function of the knee joint as an energy absorber increases, resulting in a greater amount of load experienced by the knee joint [32,35,39].

Wearing flat shoes may be a potential way to prevent and treat knee injuries. Although our study did not provide direct evidence of knee injury rates associated with flat shoes, running in flat shoes improved the variables of the hip and knee joints, which were risk factors for PFP and ITBS in prospective studies. In other words, the symptoms and functions improved following a reduction in these variables [8,9,17,18,23,24]. Previous studies also showed that running in minimalist shoes without drops and barefoot running reduced the knee extension moment and patellofemoral joint loading, thus minimizing the strain around the knee and reducing the risk of knee injuries [32,33,35,39,41]. These results provided evidence for a clinical observation made in a previous prospective study, although there was no difference in overall running−related injuries, the rate of knee injuries was 15% when wearing flat shoes, which was lower than 26% when wearing conventional shoes with a 10 mm drop [55]. Combined with the results from the literature, our results suggest that wearing flat shoes may potentially reduce runners’ risk of knee injuries. In other words, running in flat shoes increases the ankle moment during running, which may increase strains in the Achilles tendon and triceps surae muscles for runners [36]. Therefore, flat running shoes and conventional running shoes with a 10 mm drop might be beneficial for reducing specific running−related injuries, and are suitable for different people.

This study had certain limitations that should be considered. Firstly, participants were relatively young male runners. Hence, caution must be exercised when generalizing our results among female runners due to the biomechanical differences in lower limbs between sexes [56]. Secondly, only the immediate influence was analyzed in healthy runners; a long−term study involving runners with knee injuries is therefore necessary to determine the benefits of wearing flat shoes.

## 5. Conclusions

For male runners, flat running shoes result in significant decreases in contralateral pelvic drop, hip adduction, and knee internal rotation during the stance phase compared with conventional running shoes with a 10 mm drop. Given that these variables are associated with PFP and ITBS, the findings of this study may provide valuable information for reducing these running−related knee injuries.

## Figures and Tables

**Table 1:** Comparison of gait parameters and peak impact forces between flat shoes and conventional shoes with a 10 mm drop.

Variable	Flat	10 mm Drop	*p*−Value	Effect Size
Speed (m/s)	4.0 ± 0.1	4.0 ± 0.2	0.710	0.02
Step length (m)	1.47 ± 0.10	1.50 ± 0.11	0.272	0.29
Step frequency (steps/min)	164.1 ± 12.6	161.7 ± 9.6	0.304	0.21
Foot inclination angle at initial contact (°)	22.6 ± 8.5	28.3 ± 7.6	0.001	0.71
Impact peak (BW)	2.05 ± 0.40	2.03 ± 0.35	0.820	0.05
Active peak (BW)	2.75 ± 0.33	2.70 ± 0.33	0.363	0.15

Note: BW = body weight.

**Table 2:** Comparison of hip kinematics between flat shoes and conventional shoes with a 10 mm drop.

Variable	Flat	10 mm Drop	*p*−Value	Effect Size
Flexion angle at initial contact (°)	43.6 ± 8.5	44.6 ± 8.1	0.270	0.18
Adduction angle at initial contact (°)	1.8 ± 3.1	1.9 ± 2.9	0.561	0.04
Internal rotation angle at initial contact (°)	4.8 ± 5.8	2.6 ± 4.2	0.158	0.24
Peak contralateral pelvic drop (°)	6.1 ± 2.7	7.5 ± 2.8	0.003	0.51
Peak flexion angle (°)	43.4 ± 8.4	44.8 ± 8.0	0.282	0.17
Peak adduction angle (°)	13.3 ± 4.3	14.1 ± 4.7	0.161	0.18
Peak internal rotation angle (°)	9.9 ± 8.2	10.1 ± 6.5	0.837	0.03

## Data Availability

The data presented in this study are available on reasonable request from the corresponding author due to privacy.
